# The Physician’s Leisure Library

**Published:** 1888-07

**Authors:** 


**The Physician's Leisure Library.** GEO. S. DAVIS, Detroit, Publisher. Price per volume, cloth, 50c.; paper, 25c.

The publisher is to be commended for this enterprise, having for its object the publication of excellent medical works in a cheap and attractive form. We have recently received the following numbers:

NOS. 8 AND 9. THE INFECTIOUS DISEASES. By KARL LIEBERMEISTER, Professor of Internal Pathology, etc., Tuebingen, Germany.

This German medical classic has been well translated by Dr. Hurd, and we know of no other better means of acquiring a knowledge of the present methods of therapeusis in Germany than by carefully reading this work. The reader will observe that Dr. Liebermeister is a thorough believer in the bacterial origin of diseases, and his curative measures are almost entirely composed of antiseptics and antithermics. The cold bath is recommended, a remedial procedure which has never been very popular in this country. Although we do not agree with the author's conclusions in many cases, we cannot but commend the work.

No. 11. THE DISORDERS OF MENSTRUATION. By EDWARD W. JENKS, M. D., Professor of Gynecology in the Michigan College of Medicine and Surgery.

An eminently practical work for the general practitioner. It is carefully and concisely written, and contains just the information we need in the management of these troublesome disorders. We must offer one slight criticism in regard to the prescription-writing: Many of the names of drugs used are those found in pharmacopœia of 1870, and have since been changed. A new book should be up to date in every respect.

